# Spatial and Temporal Potato Intensification Drives Insecticide Resistance in the Specialist Herbivore, *Leptinotarsa decemlineata*

**DOI:** 10.1371/journal.pone.0127576

**Published:** 2015-06-01

**Authors:** Anders S. Huseth, Jessica D. Petersen, Katja Poveda, Zsofia Szendrei, Brian A. Nault, George G. Kennedy, Russell L. Groves

**Affiliations:** 1 Department of Entomology, Cornell University, New York State Experiment Station, Geneva, New York 14456, United States of America; 2 Department of Entomology, Cornell University, Ithaca, New York 14853, United States of America; 3 Department of Entomology, Michigan State University, East Lansing, Michigan 48823, United States of America; 4 Department of Entomology, North Carolina State University, Raleigh, North Carolina 27607, United States of America; 5 Department of Entomology, University of Wisconsin, Madison, Wisconsin 53706, United States of America; Rutgers University, UNITED STATES

## Abstract

Landscape-scale intensification of individual crops and pesticide use that is associated with this intensification is an emerging, environmental problem that is expected to have unequal effects on pests with different lifecycles, host ranges, and dispersal abilities. We investigate if intensification of a single crop in an agroecosystem has a direct effect on insecticide resistance in a specialist insect herbivore. Using a major potato pest, *Leptinotarsa decemlineata*, we measured imidacloprid (neonicotinoid) resistance in populations across a spatiotemporal crop production gradient where potato production has increased in Michigan and Wisconsin, USA. We found that concurrent estimates of area and temporal frequency of potato production better described patterns of imidacloprid resistance among *L*. *decemlineata* populations than general measures of agricultural production (% cropland, landscape diversity). This study defines the effects individual crop rotation patterns can have on specialist herbivore insecticide resistance in an agroecosystem context, and how impacts of intensive production can be estimated with general estimates of insecticide use. Our results provide empirical evidence that variation in the intensity of neonicotinoid-treated potato in an agricultural landscape can have unequal impacts on *L*. *decemlineata* insecticide insensitivity, a process that can lead to resistance and locally intensive insecticide use. Our study provides a novel approach applicable in other agricultural systems to estimate impacts of crop rotation, increased pesticide dependence, insecticide resistance, and external costs of pest management practices on ecosystem health.

## Introduction

Pest resistance to insecticides is an important problem in production of many crops worldwide; thus reduced insecticide efficacy in the field has practical consequences for pest control, farmer profitability, and ecosystem health [1–4]. Insecticide resistance occurs as a result of exposure of pest populations to insecticides in a host crop; and at a landscape-scale, greater abundance of uniformly treated host crops increases the selection pressure for resistance [5,6]. One tactic to control resistance development has been to separate individual crops in space and time through the use of crop rotation [5,7]. During intervals when the treated crop is not being grown, selection for resistance does not occur. Hence the frequency of selection is reduced in direct relation to the crop-rotation interval, assuming the pest is present during all these times. Moreover, if resistance is costly in terms of fitness, the benefit of crop rotation is expected to increase [5,8].

Crop rotation for resistance management is a scale-dependent tactic that can be affected by life history and dispersal ability of the pest. For highly mobile, polyphagous pests (e.g., sweetpotato whitefly, *Bemisia tabaci* Gennadius; cotton bollworm, *Heliocoverpa spp*.), resistance management plans are expected to be most effective when implemented at regional scales and could include several different target crops, insecticide inputs, and stakeholders (i.e., farmers, industry, and regulatory agencies) [9–11]. Because these pests can disperse long distances, crop rotation alone may be an inadequate tactic to reduce colonization of the crop and selection for insecticide resistance in farm fields. Yet, resistance is often slow to develop in these pests because non-agricultural or non-sprayed host environments can serve as a critical refuge for insecticide-susceptible pests that help to delay resistance development [5].

Insecticide-resistant specialist herbivores (e.g., western corn rootworm, *Diabrotica virgifera virgifera* LeConte; Colorado potato beetle, *Leptinotarsa decemlineata* Say) that do not disperse long distances can be effectively managed using crop rotation at the field- or farm-scale [7,12–15]. The link between changing crop rotation practices and specialist herbivore insecticide resistance has been documented at the scale of individual maize fields in areas of the upper Midwest where frequent maize production at the field-scale relates to resistance to a Bt maize trait (Cry3Bb1) in the specialist herbivore, *D*. *virgifera* [16].

Using *D*. *virgifera* Bt resistance estimates generated from individual maize fields in Iowa, Gassmann et al. [16] speculated that this high intensity, continuous cropping of Bt maize coupled with limited rotation of non-host crops at larger spatial scales, is one likely explanation for eroding control in the upper Midwest maize agroecosystems. Theoretical simulation studies support the link between continuous maize production and Bt resistance in *D*. *virgifera* populations at larger spatial scales [15], suggesting that selection for Bt resistance could be related to spatiotemporal maize production patterns at the landscape or regional scale. Although it is well known that homogeneous agricultural systems and uniform insecticide regimes (e.g., Bt maize and *D*. *virgifera* resistance) enable the rapid emergence of insecticide resistance [6], few empirical studies have described how changing crop composition within agricultural landscapes could affect insecticide resistance development in pest populations. Moreover, agricultural systems in which specialist herbivores have a close association to specific host crops provide a more simplified context to examine the direct effects of crop rotation on pests, pesticide use, and insecticide resistance.

Here, we tested whether field- or landscape-scale intensification (increasing spatial and temporal abundance) of the insecticides used in cultivated potato (*Solanum tuberosum* L.) has measureable, long-term effects on populations of the specialist potato pest, *Leptinotarsa decemlineata* Say (Coleoptera: Chrysomelidae), sufficient to increase the development of resistance to the neonicotinoid insecticide imidacloprid. Here, we define resistance as a genetically conferred trait that results in reduced imidacloprid susceptibility. In our definition a resistant phenotype has decreased sensitivity to neonicotinoid insecticides that has practical consequences for potato pest management. This potato-pest term extends the definition of “practical resistance” as defined by Tabashnik et al. [17] where field-evolved resistance in a pest reduces pesticide efficacy and has practical consequences for pest control. Uniform use of specific insecticides in commercial potato (i.e., pyrethroids, carbamates) has, in the past, resulted in widespread insecticide resistance in *L*. *decemlineata*, requiring additional insecticide applications, and causing significant economic loss for farmers [18]. This predisposition to insecticide resistance is a concern for potato farmers that have relied almost exclusively on neonicotinoid insecticides applied at the time of planting over the past 18 years. Prophylactic neonicotinoid use continues to occur uniformly among potato fields in the Great Lakes region of the US. Therefore, we compare landscapes with different spatial and temporal potato production patterns by holding insecticide use constant while varying potato abundance, to assess the effect these variations in potato abundance have on *L*. *decemlineata* susceptibility to neonicotinoid insecticides. As a result of uniformity of neonicotinoid use among potato farmers, we hypothesized the incidence of insecticide resistance in *L*. *decemlineata* populations would be related to abundance of neonicotinoid-treated potato in the landscape and also the frequency of its production.

## Materials and Methods

### Ethics statement

No specific permits were required for the field study described here. Access to field sites was granted by landholders to collect insects.

### *Leptinotarsa decemlineata* management and the potato agroecosystem

*Leptinotarsa decemlineata* is a specialist herbivore of plants in the family *Solanaceae*, and is an economically important potato pest in North America, Europe and Asia [19]. Neonicotinoid insecticides applied at planting are the most common approach for managing *L*. *decemlineata* in the Midwestern US. Prior to this study, data provided by the Wisconsin Potato and Vegetable Growers Association showed that a high proportion of potato area was treated with neonicotinoid insecticides for *L*. *decemlineata* control from 2003–2006 (Table 1)[20–22]. Farmers spray the neonicotinoid insecticide (i.e., clothianidin, imidacloprid, thiamethoxam) directly on potato seed pieces when the crop is planted. As the plant grows, the insecticide moves systemically through the plant xylem to leaf tissues [23,24]. Using this neonicotinoid treatment method, entire potato fields are protected from four key potato herbivores (i.e., green peach aphid, *Myzus persicae* Sulzer; potato aphid, *Macrosiphum euphorbiae* Thomas; potato leafhopper, *Empoasca fabae* Harris; Colorado potato beetle *L*. *decemlineata* Say) for nearly two months [25]. Insecticide-treated potato is often the most abundant host for *L*. *decemlineata* in temperate potato agroecosystems; however, untreated volunteer potato and several native *Solanum* weeds (e.g., nightshades, *Solanum dulcamara* L.; buffalo-bur, *Solanum rostratum* Dunal; horse-nettle, *Solanum carolinense* L.) are alternate hosts when commercial potato fields are not present [26–29]. Overwintered adult *L*. *decemlineata* walk to colonize potato fields, typically at distances less than 1.5 km from the diapause site [30]. Each season *L*. *decemlineata* completes two generations on the potato crop and diapauses as an adult in unmanaged habitats surrounding fields [31–33]. This combination of host plant specialization and short-distance dispersal makes *L*. *decemlineata* vulnerable to abundance, or paucity, of nearby potato crops and alternate hosts, and also shifts in potato pest management practices.

**Table 1:** Reported neonicotinoid use in a survey of commercial potato farmers in Wisconsin, 2003–2006.

Year	Reported area treated with neonicotinoid insecticides (ha)	Total potato area reported (ha)	Proportion treated with neonicotinoids
**2003**	**5394**	**6422**	**0.84**
**2004**	**5174**	**7392**	**0.70**
**2005**	**4953**	**6784**	**0.73**
**2006**	**4051**	**4822**	**0.84**
**Mean (±SD)**	**4893 (589)**	**6355 (1098)**	**0.78 (0.07)**

Mechanisms driving resistance development in *L*. *decemlineata* may operate at several different scales in the landscape. At the field level, a complex arrangement of landholders and farmers (many farmers rent land) has resulted in different crop management practices that contribute to variable profit expectations and, in turn, pest management decisions. All farmers used at-plant neonicotinoids as their primary pest management tactic for potato fields from which *L*. *decemlineata* populations were collected over the course of this study (4 counties in Michigan and 7 counties in Wisconsin). We also know that neonicotinoids are the predominant *L*. *decemlineata* management tool in the production region (Table 1). Although *L*. *decemlineata* resistance to neonicotinoids is an emerging problem, growers continue to spray inexpensive at-plant neonicotinoids to control the remaining pest species and gain some incidental control of *L*. *decemlineata*. Other insecticides may be used for *L*. *decemlineata* control later in the growing season; however, there is no known cross resistance between neonicotinoid and other insecticides used in this system [34, 35]. As a result of these two factors, variation in the field-level pest management practices, aside from uniformity of neonicotinoid use, will likely provide limited explanation for observed differences in *L*. *decemlineata* resistance to neonicotinoids.

Alternatively, regional-scale analyses of potato production and *L*. *decemlineata* may provide insight about the genetic predisposition for resistance in discrete geographically isolated populations [36]; however variability in resistance to individual insecticides has been previously documented to operate at smaller scales in the study region [18]. Thus far, studies of *L*. *decemlineata* resistance at either field or regional scales have not effectively described specific selection factors that influence spatial variability in *L*. *decemlineata* resistance within agroecosystems. Here we approach resistance development at the landscape scale by exploring the relationship between spatiotemporal potato production intensity and *L*. *decemlineata* resistance selection by neonicotinoid insecticides.

### *Leptinotarsa decemlineata* collection

From 2007–2012, imidacloprid susceptibility was measured at 50 locations in commercial potato production regions of Michigan and Wisconsin, USA (S1 Fig). Two large vegetable production regions include the majority of sample locations, and potato is a common component of the landscape and *L*. *decemlineata* is an annual pest of the crop. Additionally, several smaller potato production areas of Michigan and Wisconsin were sampled; these individual farms or small groups of farms also experience significant damage from *L*. *decemlineata* populations. At all locations, potato was the most abundant host crop in the environment for this specialist herbivore.

Adult *L*. *decemlineata* were collected from commercial potato fields that averaged 39±19 ha (mean±SD, min. 4, max 75) in size. The collection sites were not chosen at random; cooperators collected insects at locations where large numbers of adult insects were present. Incidence of large numbers of adult beetles may have been the result of insecticide application problems, inadequate rotation of potato, proximity to overwintering sites, or insecticide resistance [37]. Collections occurred during the month of June each year. During this period, concentration of neonicotinoid insecticides are highest in potato [38], and were the only insecticides applied to the crop. Adult insects collected during this period generally represented the overwintered generation of *L*. *decemlineata* immigrating to the potato crop.

To estimate baseline neonicotinoid susceptibility in *L*. *decemlineata*, one population was collected from insecticide-free potato outside of the WI commercial potato production region each year (2007–2012). *Leptinotarsa decemlineata* collected near Arlington, WI (hereafter called the reference population) was included as a wild population that has not been exposed to large-scale commercial potato production and associated crop inputs.

At each site, approximately 400–500 adult beetles were collected directly from potato plants into plastic cups (0.94 L). Sample numbers varied based on availability of insects in the field. Adult insects were transported to laboratories at either Michigan State University, East Lansing, MI or the University of Wisconsin-Madison, Madison, WI. Upon arrival, insects were fed insecticide-free potato foliage in screen cages maintained in environmental chambers held at 24°C and a photoperiod of 16:8 (L:D) for one week prior to bioassays to allow for mortality related to latent, chronic effects of possible insecticide exposure in the field.

### Resistance assessment

Resistance to neonicotinoids was assessed using topical imidacloprid bioassays. Technical grade imidacloprid (97.5%, Bayer Corporation, Kansas City, MO) was dissolved into pesticide grade acetone (Fisher Chemicals, Fair Lawn, NJ), then serially diluted to a range of concentrations between 0.001–10 ppm [39]. Using results of a preliminary screen of 60 randomly chosen individuals, a range of five to nine insecticide concentrations were chosen that would result in 0–100 percent mortality [39]. Adult beetles were randomly divided into equal numbers per concentration, each containing no fewer than fifteen insects per concentration. Collected individuals were topically treated with one microliter of insecticide solution applied to the first abdominal sternite with a repeating dispenser equipped with a 50 μL blunt syringe (PB-600 and 700 series, Hamilton Company, Reno, NV). Control insects received a one microliter dose of pesticide-grade acetone alone. Treated insects were placed into 100x15 mm Petri dishes with filter paper (Fisher Scientific, Pittsburgh, PA) and maintained on insecticide-free potato foliage in an environmental chamber held at 24°C and a photoperiod of 16:8 (L:D). Bioassay response was measured at day seven post-treatment. Insects were classified as alive, intoxicated, or dead. Intoxicated beetles were unable to grasp the tip of a wooden pencil and walk greater than one body length up the pencil [39]. Because prior research showed intoxicated *L*. *decemlineata* do not recover and reproduce, intoxicated and dead insects were pooled for subsequent statistical analyses [39].

Imidacloprid bioassay dose-response results were first adjusted for control mortality using Abbott's correction [40], and then analyzed against imidacloprid concentration with a Log_10_ probit regression analysis in SAS [41, 42]. Goodness of fit chi-square tests were used to interpret satisfactory performance of model fitting and differences of within population susceptibility [43]. Fifty-percent lethal concentration estimates (LC_50_) were calculated to determine the relative imidacloprid susceptibility of populations. Presented and analyzed here are fifty-percent lethal concentration estimates to compare the average response of different *L*. *decemlineata* populations to imidacloprid.

### Agricultural landscapes

To determine the agroecosystem composition surrounding sampled *L*. *decemlineata* populations, sample fields were digitized from orthorectified aerial imagery [44], then converted to a point representing the field centroid in ArcGIS (Version 10.1, ESRI, Redlands, CA). Semi-natural and crop habitats were quantified within 1.5 km of field centroids, the maximum dispersal distance documented for *L*. *decemlineata* (Buffer tool, ArcGIS) [30]. Data describing the composition of managed agricultural and semi-natural habitats surrounding sampled fields were derived from publicly available, National Agricultural Statistics Service—Cropland Data Layer [45]. The data layer is a remotely sensed, categorical description of crop and semi-natural land using georeferenced 30x30m squares (raster pixels) that can be used to measure spatial patterns of crop production [45]. The bioassay crop year was used to quantify the amount of cultivated agriculture and potato in the landscape (S2a Fig). Crops included in the landscape were forages, fruit, maize, pea, potato, dry beans, small grains, vegetables, and other miscellaneous crops (S2b Fig).

Potato crop history was determined from four sequential years prior to each bioassay. Land cover data were reclassified into a binary layer (potato or not) then sequentially summed to generate four potato crop sequences: one, one and two, one through three, and one through four consecutive prior years of potato production (Raster Calculator, ArcGIS). Land cover and potato raster data for each bioassay location and prior crop year combination were extracted and tabulated using Python and the integrated ArcPy site package (version 2.7.2).

To examine the broader effects of landscape composition, land use raster data within each 1,500 m buffer around each field from each bioassay year were used to measure one common estimate of landscape pattern, Shannon’s Diversity Index (SHDI) [46]. Landscape compositions including cultivated cropland and semi-natural habitats were used to calculate SHDI using FragStats [47]. Although several other measures of landscape composition exist, SHDI represents one easily interpretable metric that is likely correlated to other metrics of landscape pattern [48].

### Statistical analysis

**Fig 1:**
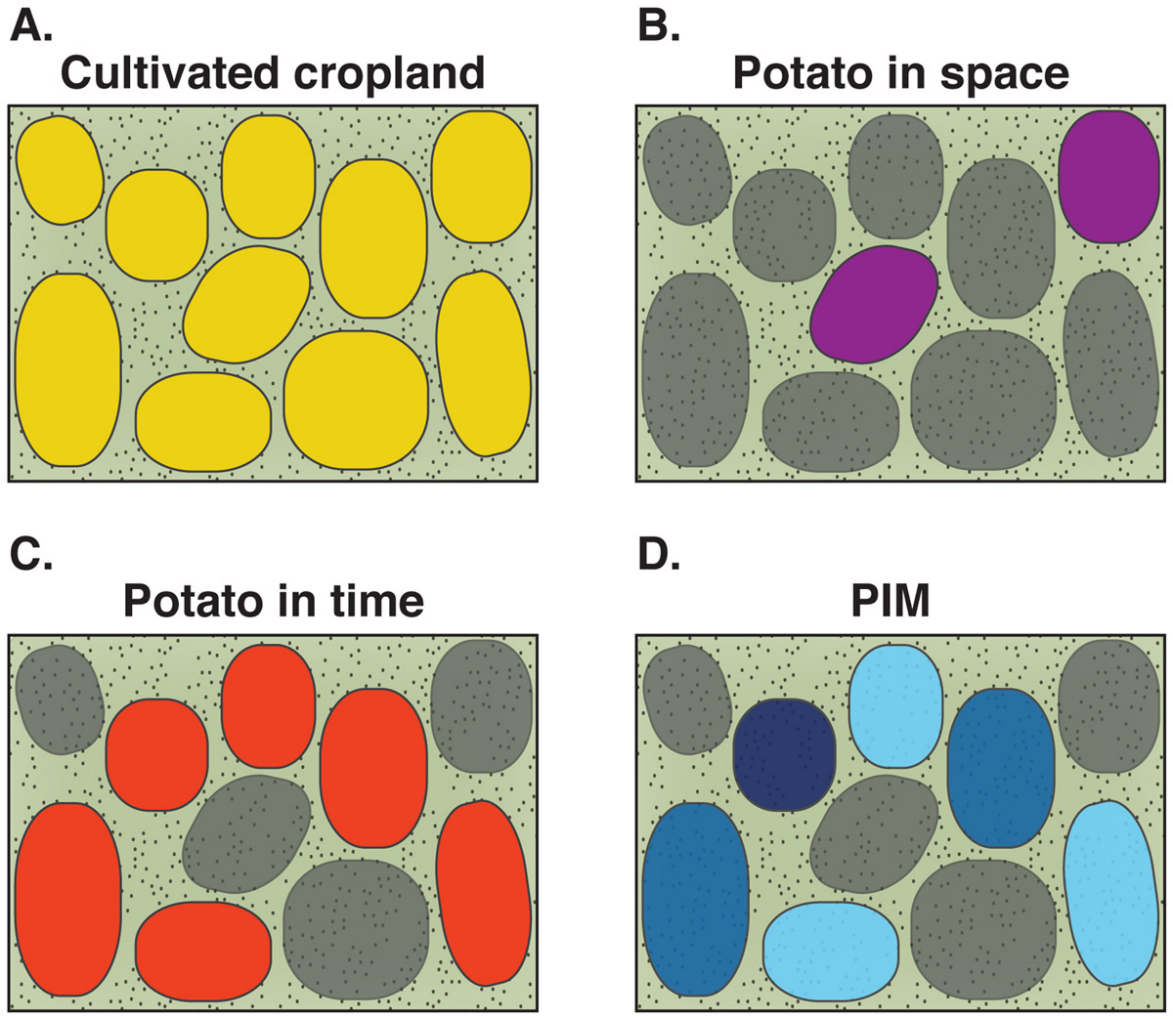
Conceptual diagram of cultivated cropland and potato measurements. **(a)** Yellow fields represent the area of cultivated cropland in the landscape. Cultivated cropland was divided by the total landscape area to measure the proportion of the ecosystem used for agriculture. **(b)** Purple fields represent the available cropland used for potato production in the year *L*. *decemlineata* populations were collected. Proportion current potato was calculated by dividing area of purple fields by area of yellow fields. **(c)** Red fields represent the area of cropland used for potato production in at least one of the four years preceding the *L*. *decemlineata* collection year for bioassays. Proportion potato in time was calculated by dividing the area of red fields by area of yellow fields. **(d)** The gradient of blue colored fields represents the frequency of potato production on fields that had historically been potato in one to four years before the bioassay. Light to dark blue represents an increase in planting frequency over years. Each of these areas was used to calculate the potato intensity metric (PIM).

A linear modeling approach was used to determine the relationship between *L*. *decemlineata* resistance (LC_50_ estimates) and proportion cultivated agriculture in the landscape (Fig 1a), proportion of available cropland used for potato production in the bioassay year (potato cultivation over space) (Fig 1b), proportion of available cropland used for potato production over the potato intensity interval (potato cultivation over time)(Fig 1c), and potato intensity metric (PIM)(Fig 1d). The potato intensity metric was calculated as follows:

$$PIM=\frac{\sum_{i=1}^{j}•T_{i}}{Potato\;area_{i}}$$

where *i* is a year prior to resistance sampling and *j* is every consecutive year prior to *i*. *T*_*i*_ is the total area (number of pixels) of potato in the landscape for each of the years prior to resistance sampling. *Potato area*_*i*_ is the total area (number of pixels) used for potato in any of the four years prior to resistance sampling. Because the PIM incorporates both spatial and temporal potato production into a single metric, differentiating effects of either space or time becomes challenging. To account for this interaction, we chose to include models that included only abundance of potato over four years (potato cultivation over time) to estimate the effect of patch abundance over the PIM interval. Although *L*. *decemlineata* is a concern for farmers, pest pressure is only a minor component of potato planting decisions. Several other factors (e.g., soil pathogens, fuel costs) also influence the location of fields in the landscape. To our knowledge, farmers do not bias planting decisions in space based on prior *L*. *decemlineata* management records, pest pressure, or anecdotal estimations of *L*. *decemlineata* resistance to neonicotinoids. Therefore, relationships do not indicate simultaneous causality between resistance and history of potato production.

We observed a difference between estimated LC_50_ for imidacloprid by state (S3 Fig); this state-level difference has been consistent for many previously used insecticide modes of action [37,49]. An additional explanation for this difference could be operational differences among labs conducting bioassays; however, we minimized this possibility by standardizing protocols for solution preparation, dose selection, growth chamber conditions, and bioassay scoring. To account for unmeasured population-level differences (e.g., genetic, pest management history, laboratory) between states, a state factor and resulting interactions with predictors were included in all saturated models. Preliminary analysis found that SHDI and proportion cultivated cropland in the landscape were significantly correlated (*r* = -0.28, t_(48)_ = -2.054, *P* = 0.04). We chose to model proportion cultivated cropland only. All linear regressions were completed in R using *lm* in the base package [50]. All functions used in R are denoted in italics. Homogeneity of the error variance between states was examined with Levene’s test using group medians [51]. There was no evidence of inconsistent variance between states (*F*_(1,48)_ = 0.49, *P* = 0.49). Model fits were examined for patterns in residual distribution and deviations from assumptions of normality [52]. Sensitivity of ordinary least-squares regression models to possible outliers was determined using the Bonferroni correction method for points with the largest residual values [51]. Presence of influential observations was examined with Cook’s statistics, half-normal plots, and added variable plots [53]. Model diagnostic assessments did not justify the removal of any outlier or highly influential points.

Candidate models tested combinations of the potato in space, potato in time, PIM, or proportion agriculture parameters. Models tested each parameter individually with a fixed effect for state and also an interaction with state. All possible combinations of parameters were also tested (N = 30 possible models). Parameters in nested models were eliminated using a backward selection strategy with ANOVA and sequential *F*-tests using the *drop1* function. No higher-level interactions were tested beyond the covariate of interest by state interaction. Non-nested models were sequentially ranked from lowest to highest score and all candidate models with an AIC score within four of the best models are presented. If two competing models were within an AIC score of two, main effect and interaction terms that were not significant were eliminated with the *drop1* function (test = “*F*”). All parameter estimates, standard errors, and model fit diagnostics for the final model were generated with the *summary* function. Data were transformed (Log_10_) to meet the assumptions of normality. Model fit predictions were generated with the *predict* function. Correlation between PIM, SHDI, and proportion cultivated cropland in the landscape were done with *cor*.*test* function [51].

## Results and Discussion

**Fig 2:**
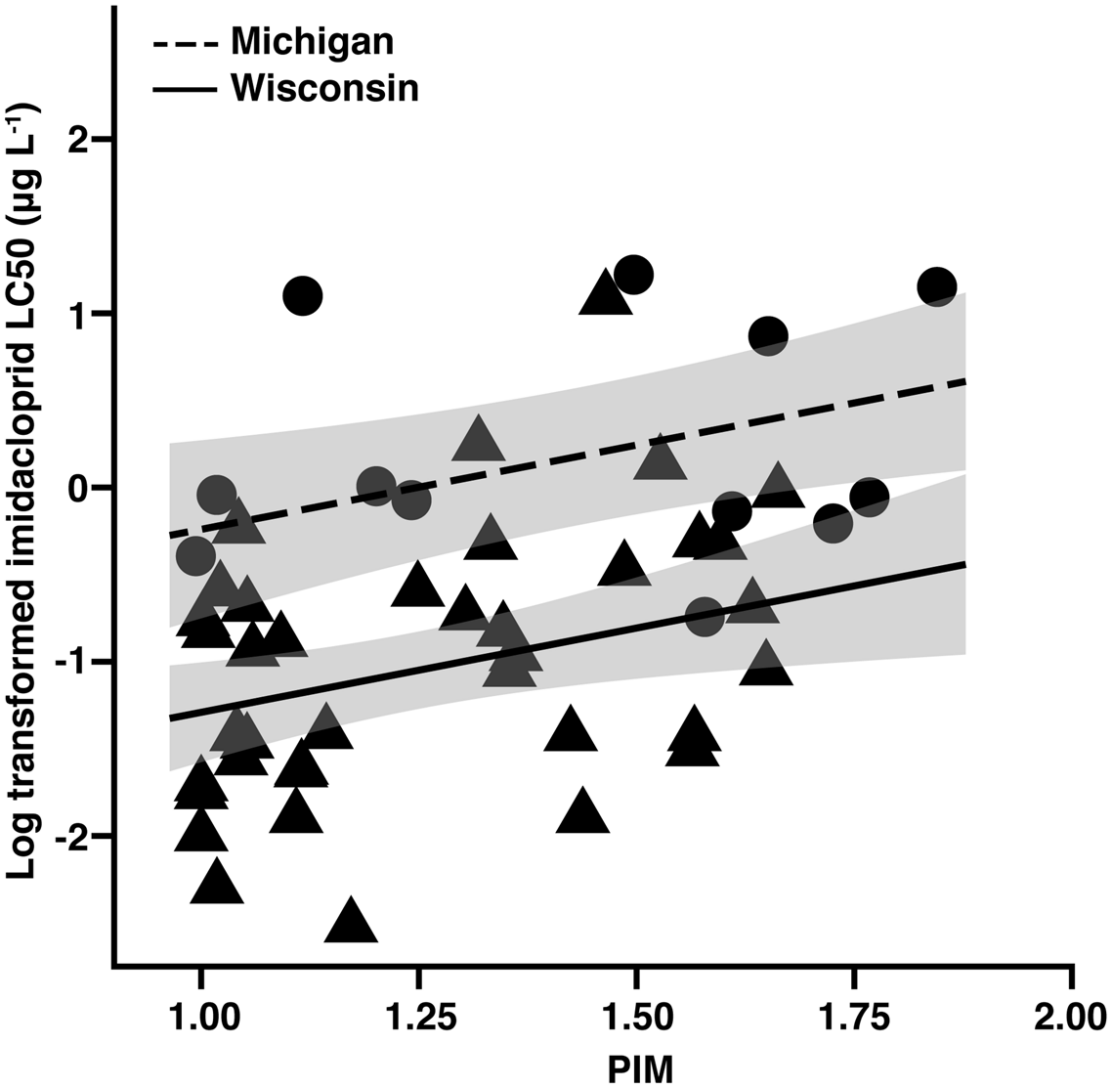
Potato intensity and imidacloprid resistance. Potato intensity metric (PIM) is log-linearly, positively related to the incidence of imidacloprid resistance in sampled populations of *L*. *decemlineata* (N = 50 populations). PIM is a metric that accounts for both area and history of potato production. Shading illustrates 95% confidence intervals of the mean. Circle (●) data points represent Michigan and triangles (▲) represent Wisconsin.

Using PIM, we evaluated the link between spatiotemporal crop production and insecticide resistance. There was a significant positive relationship between PIM and imidacloprid resistance in *L*. *decemlineata* (*F*_(2,47)_ = 17.72, *P*<0.01, R^2^ = 0.43; Fig 2; S1 Table); this positive relationship shows increasing potato intensity was associated with an increase in insecticide resistance (Fig 2). Uniformity of neonicotinoid use among potato fields (Table 1) further supports the conclusion that greater abundance of potato in space and time (PIM) could increase exposure to neonicotinoid insecticides, thereby driving imidacloprid resistance in *L*. *decemlineata*. We also found that the proportion of cultivated cropland in the surrounding environment did not significantly relate to the measured level of neonicotinoid resistance in *L*. *decemlineata* (Table 2). This relationship shows that some *L*. *decemlineata* populations were isolated from other agricultural production and also had high levels of resistance.

**Table 2:** Parameter estimates (±SE), AIC and difference in AIC from best models fitting log transformed LC_50_ estimates of resistance in *L*. *decemlineata* populations.

model parameters^a^	intercept	PIM	% potato in space^b^	% potato in time^c^	% cultivated cropland	state_*wisconsin*_	Interaction term ^d^	AIC	Δ AIC
**PIM+state**	-1.28* (0.62)	1.05* (0.41)				-1.05* (0.24)		110.666	-
PIM*state	-0.28 (1.02)	0.35 (0.69)				-2.51* (1.20)	1.06 (0.85)	111.028	0.361
% cropland*state	1.77 (1.19)				-2.65 (2.02)	-3.54* (1.25)	3.98 (2.11)	112.780	2.114
**% potato in time+state**	-0.34 (0.34)			0.86* (0.41)		-1.12* (0.24)		112.804	2.137
% potato in time*state	-0.01 (0.56)			0.35 (0.80)		-1.55* (0.63)	0.69 (0.93)	114.217	3.551
**% cropland+state**	-0.35 (0.41)				0.99 (0.61)	-1.23* (0.24)		114.493	3.826
**% potato in space+state**	-0.08 (0.31)		1.06 (0.79)			-1.19* (0.24)		115.329	4.662

^a^ Parameter estimate differs significantly from zero (*, *P* < 0.05)

^b^ Proportion potato grown on available cropland in the year bioassays were conducted

^c^ Proportion cultivated cropland where potato was grown at least once in the four years preceding the bioassay

^d^ Represents the interaction between specified model parameter and state

There was no significant relationship between *L*. *decemlineata* resistance and proportion of cultivated cropland in the ecosystem. We found that average imidacloprid LC_50_ of the reference population was 0.04±0.02 μg L^-1^ (mean±SD; min. 0.02; max. 0.09) over six consecutive years. This estimate represents baseline tolerance of a population not exposed to commercial potato or the insecticides used in that crop. In comparison, insects collected from landscapes where potato was grown had higher estimates of neonicotinoid resistance with an average imidacloprid LC_50_ of 0.74±0.81 μg L^-1^ (mean±SD; min. 0.08; max. 3.39), which was a 19-fold (range 4 to 37 fold) difference in resistance.

**Fig 3:**
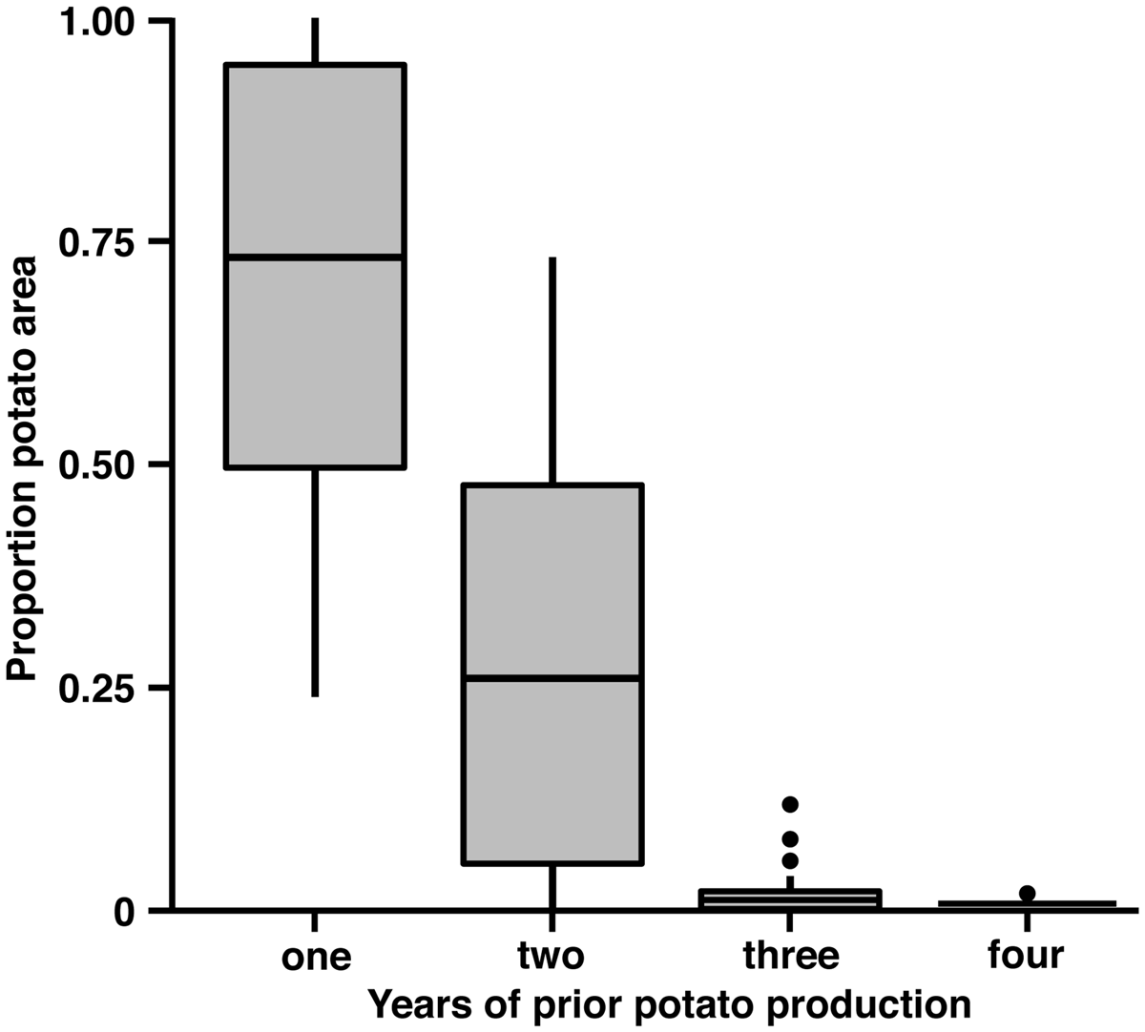
Potato production history. Potato incidence on cultivated cropland over four consecutive years of prior production estimated from within a 1.5 km radius surrounding each sample field centroid (N = 50 fields). Frequency of production shows that farmers often rotate potato at variable time intervals, ranging from low intensity production (potato occurring once in four years) to high intensity production (continuous potato).

One explanation for differences in *L*. *decemlineata* resistance levels between the reference population and other sample sites could be the amount of insecticides used in agriculture surrounding collection locations. In a regional study of insecticide inputs, amount of insecticides and pest pressure in this region were positively correlated to proportion cultivated cropland in the landscape [54]. To determine if the landscape structure surrounding the reference population could have affected the low estimates of neonicotinoid resistance, we measured the composition of cultivated cropland over the six-year collection interval. We found that 0.55±0.25 (mean proportion±SD, min. 0.08, max 0.99) of available land in agricultural production was used to grow potato in at least one of the four prior growing seasons (Fig 3). The dominant landscape matrix surrounding the reference population was 0.72±0.04 proportion cultivated cropland (mean proportion±SD, min. 0.62, max. 0.75). Potato was a very minor component in the landscape with less than a hectare grown each season. In contrast, the proportion cultivated cropland surrounding *L*. *decemlineata* populations collected from commercial potato production areas was more variable 0.57±0.17 (mean proportion±SD, min. 0.20, max. 0.90). Proportion cultivated cropland did not show a clear relationship to *L*. *decemlineata* resistance for populations collected in agroecosystems where potato is grown commercially. Moreover, the reference population was collected from a landscape dominated by cultivated cropland, yet the reference population was, on average, 19-fold more susceptible to imidacloprid on average compared with than populations collected where commercial potato was grown. This comparison further supports the hypothesis that production intensity of potato in the landscape influences resistance development in *L*. *decemlineata*.

The proportion of cultivated cropland and PIM were not significantly correlated (*r* = 0.19, t_(48)_ = 1.336, *P* = 0.19), at the 1.5 km spatial scale (S4 Fig). Predictions of other studies [54, 55] suggest intensively managed crops, such as potato, would tend to occur in landscapes dominated by agriculture. For *L*. *decemlineata*, the type of crops grown on available farmland in space and time mattered much more than the quantity of agricultural production in the landscape. Because fields containing resistant populations were not consistently found in agriculturally dominated landscapes, we expected that the amount of land devoted to potato production would have a larger effect on insecticide resistance than the proportion of the total landscape in agricultural production. We found that the proportion of cultivated cropland occupied by potato in at least one of the prior four years was significantly related to measured resistance in *L*. *decemlineata* populations (*F*_(2,47)_ = 15.98, *P*<0.01, R^2^ = 0.40). Independent measures of potato in space or potato in time did not describe *L*. *decemlineata* resistance as well as PIM, which simultaneously accounts for a spatial and a temporal crop production component (Table 2). The significance of both potato production in time and PIM indicates that landscape measurements using a singular spatial response may be strengthened with a temporal abundance component. These results further suggest that continued displacement of other crops by potato in time and space would result in more widespread neonicotinoid resistance.

**Fig 4:**
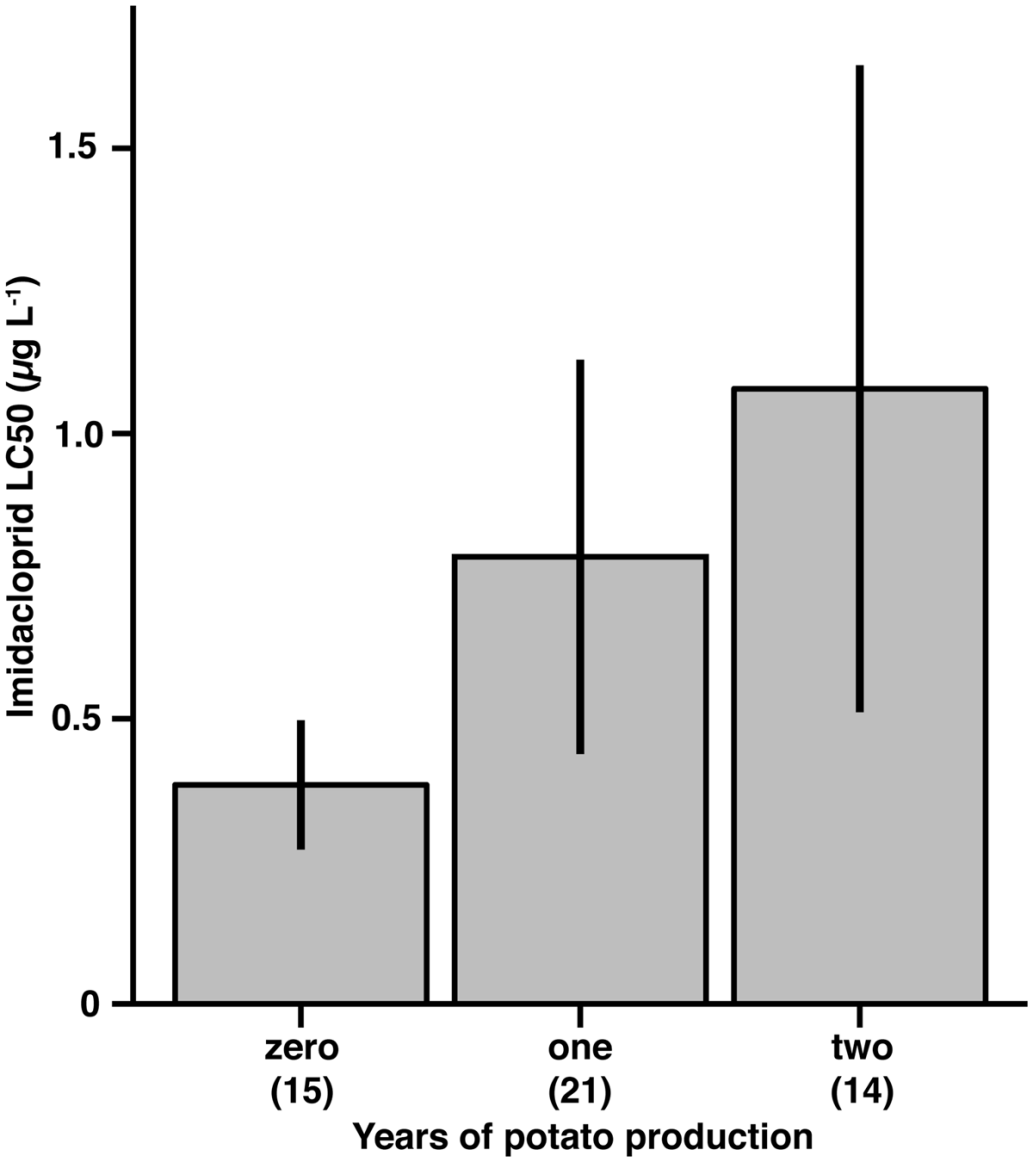
Potato production history and imidacloprid resistance. Average lethal concentration responses of *L*. *decemlineata* populations to imidacloprid compared to frequency of potato production in bioassay collection fields (N = 50 populations). Years of potato production indicate the number of potato crops grown during the four years preceding the bioassay year. Error bars represent 95% confidence intervals of the mean. Numbers in parentheses represent the count of fields in each group.

To better understand crop rotation decisions of potato farmers, we measured main crop groups (beans, forage, maize, potato, small grains, vegetables) grown in a four-year sequence in fields where *L*. *decemlineata* populations were collected. There were 45 different crop rotation combinations for the 50 fields; however, farmers chose to plant maize or potato in 115 of 200 possible crop rotation decisions (57.5%) (S5 Fig). Moreover, in the prior four years of production, 14 fields grew potato twice (28%), 21 only once (42%), and 15 did not produce potato in any other year (30%). Differences in mean LC_50_ showed a marginally significant, positive effect of more frequent potato production on resistance estimates (*F*_(2,47)_ = 2.802, *P* = 0.07; Fig 4).

Our results demonstrated the importance of abundance of an insecticide-treated crop in the landscape coupled with the frequency of its production (e.g., PIM) at a landscape scale when designing resistance management strategies for specialist insect pests. Although this crop-pest-resistance interaction is logical, we could find no studies that have documented this spatiotemporal crop relationship at the landscape scale, and likely the strength of this effect is influenced not only by crop intensity but also pest life history, dispersal distance, natural enemy control, and host range. In other systems, quantitative tools used to describe selection factors for resistance development in landscapes will need to fit the crops, individual pests, and production practices (i.e., insecticide use) of the agroecosystem where they are found.

Our spatiotemporal approach used publicly available, remotely sensed crop data to generate one simple estimate of potato intensification that was associated with measured levels of *L*. *decemlineata* resistance at the landscape scale. This analysis confirmed our potato intensity hypothesis: the level of insecticide resistance in *L*. *decemlineata* populations was positively related to abundance of insecticide-treated potato in the landscape and also the frequency of its production (PIM). Furthermore, this approach defines effects of landscape composition on a more biologically meaningful scale encompassing both spatial and temporal dimensions than approaches only measuring proportion cultivated cropland or proportion potato in a single season [55]. While proportion cropland may be a suitable predictor for pest responses in a monoculture system of a few crops, it may not consistently describe specific crop-pest interactions in more diverse production systems where many crops are grown. Sustainable intensification of agriculture will require an increase in crop diversity to reduce input reliance [56,57], a transition that will demand more flexible tools to assess landscape-scale questions about individual crops in diverse agroecosystems.

This reasonable association between insecticide resistance selection and abundance of any specific crop may not hold for more polyphagous herbivores that are exposed to variable mosaics of many host crops, insecticides, and pesticide-free refugia in the ecosystem. While other studies have focused on spatial composition of agriculture in landscapes [54,55], the findings of this study build on those results by identifying the importance of both spatial and temporal scales when examining indirect relationships between the proportion of cultivated cropland in the ecosystem, insecticide use, and negative impacts on specialist pest populations.

This crop-specific, spatiotemporal approach using remotely sensed crop cover data could have a broad range of applications to measure impacts of agricultural intensification on other pests. In temperate, conventional production systems, farmers have increased the frequency of crop production (e.g., bean, cotton, maize) in response to improved precision agriculture techniques, genetic modification (GM) technology, and increasing costs of fossil fuels [58,59]. As a direct result of these changes in production, the composition of some agricultural landscapes has rapidly transitioned from diversified crop rotations to entire regions dominated by a few profitable crops grown at a high frequency [45,56]. This intensification process improves yields by reducing pest damage via advanced agricultural technologies that reduce production amendments over large areas (GM insect control replacing conventional insecticides) [59,60]. Although advocates may argue this reduction in total inputs per unit area combined with near universal adoption of a specific technology can be an effective method to sustainably intensify production on existing farmland [61], this large-scale intensification strategy in U.S. maize production has likely contributed to the development of Bt maize resistance in the specialist herbivore, *D*. *virgifera* [16,58]. Similar patterns of resistance are likely to develop where pest management practices are adopted over large geographic extents and used frequently in time, a process that may decrease the overall sustainability of agricultural production.

Proactive insecticide resistance management programs are critical to effective insecticide stewardship from the field to the global scale [62]. While approaches to managing pest resistance may vary across pests, crops, and geographic regions, patterns of increasing insecticide use (i.e., spray frequency, higher rates, multiple insecticides) are often reflective of changing sensitivity in a pest population [11]. Unfortunately, timely insecticide input records are not easily obtained at a scale that is meaningful in a pest and crop production context. As a result, researchers tend to monitor high-risk pest species to detect developing resistance to insecticides in major crops (e.g., maize, rice, bean, cotton) [62], an exercise that does little to change pest management practices of individual farmers or improve the sustainability of minor crops in a meaningful timeframe.

Here, we used potato data to develop a metric that is specific enough to assess field-scale potato intensification to proactively manage *L*. *decemlineata* neonicotinoid resistance. Furthermore, this approach could be adapted to generate risk assessments for other resistant specialist pests for which spatial and temporal crop abundance data exists. Since 2008, the USDA NASS has produced annual CDL data spanning the contiguous US and containing a georeferenced inventory of more than 100 categorical crop classes [45]. Application of this information to other intensified crop systems will be a powerful tool to understand the effect of crop rotation on pest management at an agroecosystem, regional, or national context. Furthermore, estimates of crop intensification are likely correlated to many other inputs used annually (e.g., fungicides, herbicides, irrigation, nutrients), and could serve as a landscape- or regional-scale predictor for overall environmental risk posed by increasing production intensity of specific crops grown in monoculture or within more diverse agricultural landscapes.

In an increasingly globalized agricultural community, farmers will have greater access to similar, technologically sophisticated approaches (e.g., crop-protection products, insect resistant and herbicide tolerant GM crops) to intensify individual, high-value crops; perhaps at the cost of eliminating other ‘low-tech’ insecticide resistance management strategies, such as crop rotation. Anticipated technological advancement of global agriculture will increase the importance of proactive strategies that reduce chances of pest and disease outbreaks, crop failure, reduced yields, or negative social and environmental externalities [63–65]. For the specific conditions of our study (i.e., a specialist herbivore with limited dispersal capabilities in a system of pervasive and uniform technology adoption), our results demonstrate that combinations of spatial and temporal crop production patterns are important to describe the indirect costs of technology adoption to intensify agriculture, and should be considered in the design of strategies to achieve more sustainable methods that improve productivity of global agriculture.

## Supporting Information

S1 Dataset Potato production data and aggregated landscape data. Potato area columns represent the prior years of production (ha) prior to measurement of *L*. *decemlineata* resistance. Total potato area column represents the sum of those areas. Proportion data were calculated from data included in Dataset 2. Proportion agricultural area in potato was calculated as the difference between total area of potato production and area of total cultivated agriculture area.
(DOCX)

S2 Dataset Area of land cover (ha) surrounding *L*. *decemlineata* populations sampled in the bioassay year. Land cover estimates were tabulated from NASS CDL data using a 1.5 km buffer surrounding sample field centroids.
(DOCX)

S1 Fig Distribution of *L*. *decemlineata* populations. Map of *L*. *decemlineata* populations (N = 50) assayed for neonicotinoid resistance from 2007 to 2012. At each location, adult *L*. *decemlineata* were sampled from commercial potato fields and exposed to a dose-response bioassay. Circle (●) data points represent collections at commercial potato fields and the triangle (◼) represents reference population collection.
(TIF)

S2 Fig Average land cover composition within 1.5 km of sample fields. Distribution of major land cover types **(a)** comprising average proportion of 1.5 km radius surrounding sample field centroid. Distribution of dominant agricultural crop types comprising the average proportion cropland **(b)** of 1.5 km radius surrounding sample field centroid. Land cover compositions were measured in each year of *L*. *decemlineata* bioassay (N = 50 fields). Minor crops (i.e., fruit, miscellaneous crops, pea, small grains, and other vegetables) were aggregated for graphical presentation.
(TIF)

S3 Fig Average *L*. *decemlineata* response to imidacloprid. Average lethal concentration responses of *L*. *decemlineata* beetle populations to imidacloprid by state from 2007 to 2012. Error bars represent standard deviation of means. Lethal concentration estimates were significantly different between states (Student’s t-test, *t* = 3.2589, df = 12.467, *P* = 0.0065).
(TIF)

S4 Fig Potato intensity and proportion cropland within 1.5 km of sample sites. Relationship between the potato intensity metric (PIM) and proportion cropland in the landscape (N = 50 fields). Measures of proportion cropland and PIM were not significantly correlated.
(TIF)

S5 Fig Crop composition within 1.5 km of sample sites. Frequency distribution of major crop groups grown in four prior seasons at 50 different field locations where *L*. *decemlineata* populations were assayed for neonicotinoid resistance from 2007 to 2012. Numbers in parentheses indicate the percentage of total counts for each group.
(TIF)

S1 Table Regression coefficients for final model relating neonicotinoid insecticide resistance to the potato intensity metric (PIM). (DOCX)
