# Supplementary material for: Spatial and Temporal Potato Intensification Drives Insecticide Resistance in the Specialist Herbivore, Leptinotarsa decemlineata
Source: PLoS One. 2015 Jun 1;10(6):e0127576. doi: 10.1371/journal.pone.0127576 (PMC4452079; doi:10.1371/journal.pone.0127576)
Supplement: S1 Dataset — Potato area columns represent the prior years of production (ha) prior to measurement of L. decemlineata resistance. Total potato area column represents the sum of those areas. Proportion data were calculated from data included in Dataset 2. Proportion agricultural area in potato was calculated as the difference between total area of potato production and area of total cultivated agriculture area. (DOCX) [file pone.0127576.s001.docx]

| **ID** | **State** | **LD_50_** | **PDM^*^** | **Sample field area** | **Years of potato** | **Potato area 1 y** | **Potato area 2 y** | **Potato area 3 y** | **Potato area 4 y** | **Total potato area in 4 y** | **Total cultivated agriculture area^†^** | **Proportion cultivated agriculture** | **Proportion natural area** | **Proportion urban land use** | **Proportion potato in space^‡^** | **Proportion potato in time^§^** |
| --- | --- | --- | --- | --- | --- | --- | --- | --- | --- | --- | --- | --- | --- | --- | --- | --- |
| 1 | MI | 0.93 | 1.248 | 7 | 0 | 158.3 | 37.5 | 6.3 | 0.0 | 202.1 | 359.9 | 0.51 | 0.43 | 0.06 | 0.20 | 0.56 |
| 2 | MI | 3.01 | 1.123 | 45 | 1 | 180.8 | 21.7 | 1.7 | 0.0 | 204.2 | 368.6 | 0.52 | 0.42 | 0.06 | 0.27 | 0.55 |
| 3 | MI | 0.48 | 1.583 | 15 | 2 | 201.9 | 263.4 | 5.8 | 0.2 | 471.2 | 551.0 | 0.77 | 0.18 | 0.05 | 0.54 | 0.86 |
| 4 | MI | 2.39 | 1.657 | 54 | 2 | 161.9 | 294.7 | 4.1 | 0.0 | 460.7 | 503.7 | 0.71 | 0.23 | 0.06 | 0.47 | 0.91 |
| 5 | MI | 3.39 | 1.503 | 65 | 2 | 117.6 | 116.8 | 0.7 | 0.0 | 235.2 | 315.3 | 0.44 | 0.49 | 0.06 | 0.46 | 0.75 |
| 6 | MI | 0.87 | 1.616 | 26 | 2 | 98.9 | 154.1 | 1.3 | 0.0 | 254.3 | 357.0 | 0.50 | 0.45 | 0.04 | 0.06 | 0.71 |
| 7 | MI | 1.01 | 1.207 | 14 | 2 | 97.3 | 25.3 | 0.1 | 0.0 | 122.7 | 430.2 | 0.60 | 0.39 | 0.00 | 0.12 | 0.29 |
| 8 | MI | 0.96 | 1.024 | 43 | 1 | 161.2 | 3.6 | 0.2 | 0.0 | 165.0 | 333.7 | 0.47 | 0.50 | 0.03 | 0.14 | 0.49 |
| 9 | MI | 3.16 | 1.825 | 36 | 2 | 67.8 | 191.7 | 21.9 | 1.9 | 283.3 | 394.5 | 0.55 | 0.41 | 0.04 | 0.37 | 0.72 |
| 10 | MI | 0.81 | 1.730 | 57 | 2 | 112.0 | 270.6 | 7.1 | 0.2 | 389.8 | 392.4 | 0.55 | 0.39 | 0.06 | 0.36 | 0.99 |
| 11 | MI | 0.95 | 1.772 | 21 | 1 | 110.4 | 326.6 | 9.1 | 0.2 | 446.3 | 480.4 | 0.68 | 0.37 | 0.05 | 0.33 | 0.93 |
| 12 | MI | 0.68 | 1.000 | 75 | 0 | 48.3 | 0.0 | 0.0 | 0.0 | 48.3 | 482.7 | 0.68 | 0.26 | 0.05 | 0.14 | 0.10 |
| 13 | WI | 0.46 | 1.001 | 27 | 1 | 132.2 | 0.2 | 0.0 | 0.0 | 132.4 | 366.1 | 0.51 | 0.40 | 0.09 | 0.26 | 0.36 |
| 14 | WI | 0.41 | 1.092 | 60 | 0 | 204.0 | 20.4 | 0.1 | 0.0 | 224.5 | 410.2 | 0.58 | 0.33 | 0.10 | 0.39 | 0.55 |
| 15 | WI | 0.14 | 1.000 | 63 | 0 | 117.4 | 0.0 | 0.0 | 0.0 | 117.4 | 408.2 | 0.57 | 0.38 | 0.05 | 0.26 | 0.29 |
| 16 | WI | 0.50 | 1.053 | 54 | 0 | 128.6 | 7.2 | 0.0 | 0.0 | 135.8 | 413.6 | 0.58 | 0.30 | 0.12 | 0.30 | 0.33 |
| 17 | WI | 0.43 | 1.008 | 20 | 0 | 358.9 | 3.0 | 0.0 | 0.0 | 361.9 | 562.2 | 0.79 | 0.12 | 0.09 | 0.19 | 0.64 |
| 18 | WI | 0.19 | 1.115 | 30 | 0 | 132.5 | 17.3 | 0.0 | 0.0 | 149.7 | 350.1 | 0.49 | 0.31 | 0.20 | 0.16 | 0.43 |
| 19 | WI | 0.24 | 1.041 | 58 | 0 | 118.4 | 5.0 | 0.0 | 0.0 | 123.5 | 610.7 | 0.86 | 0.07 | 0.07 | 0.14 | 0.20 |
| 20 | WI | 0.17 | 1.000 | 33 | 0 | 49.4 | 0.0 | 0.0 | 0.0 | 49.4 | 164.2 | 0.23 | 0.59 | 0.18 | 0.22 | 0.30 |
| 21 | WI | 0.79 | 1.043 | 26 | 1 | 261.9 | 11.2 | 0.3 | 0.0 | 273.4 | 370.6 | 0.52 | 0.40 | 0.08 | 0.42 | 0.74 |
| 22 | WI | 0.24 | 1.144 | 14 | 1 | 289.8 | 47.3 | 0.6 | 0.0 | 337.7 | 396.8 | 0.56 | 0.37 | 0.07 | 0.23 | 0.85 |
| 23 | WI | 0.21 | 1.046 | 28 | 0 | 244.6 | 11.0 | 0.4 | 0.0 | 255.9 | 363.5 | 0.51 | 0.36 | 0.13 | 0.08 | 0.70 |
| 24 | WI | 0.23 | 1.053 | 36 | 0 | 260.2 | 14.5 | 0.0 | 0.0 | 274.6 | 441.4 | 0.62 | 0.27 | 0.11 | 0.18 | 0.62 |
| 25 | WI | 0.15 | 1.109 | 33 | 1 | 92.5 | 11.3 | 0.0 | 0.0 | 103.8 | 348.0 | 0.49 | 0.42 | 0.09 | 0.12 | 0.30 |
| 26 | WI | 0.35 | 1.354 | 27 | 1 | 195.5 | 89.8 | 6.8 | 0.0 | 292.1 | 465.4 | 0.65 | 0.25 | 0.09 | 0.08 | 0.63 |
| 27 | WI | 0.19 | 1.114 | 27 | 1 | 234.1 | 27.8 | 1.2 | 0.0 | 263.0 | 401.8 | 0.56 | 0.36 | 0.08 | 0.40 | 0.65 |
| 28 | WI | 0.44 | 1.347 | 20 | 1 | 326.6 | 164.6 | 3.5 | 0.0 | 494.8 | 587.4 | 0.82 | 0.09 | 0.08 | 0.24 | 0.84 |
| 29 | WI | 0.55 | 1.022 | 57 | 2 | 236.0 | 5.3 | 0.0 | 0.0 | 241.3 | 501.5 | 0.70 | 0.19 | 0.10 | 0.32 | 0.48 |
| 30 | WI | 0.15 | 1.438 | 27 | 2 | 175.8 | 114.7 | 8.1 | 0.0 | 298.5 | 493.5 | 0.69 | 0.25 | 0.05 | 0.47 | 0.60 |
| 31 | WI | 0.10 | 1.018 | 42 | 1 | 19.5 | 0.4 | 0.0 | 0.0 | 19.9 | 240.2 | 0.34 | 0.59 | 0.07 | 0.14 | 0.08 |
| 32 | WI | 0.24 | 1.424 | 24 | 2 | 28.9 | 18.9 | 0.9 | 0.0 | 48.7 | 262.9 | 0.37 | 0.59 | 0.04 | 0.08 | 0.19 |
| 33 | WI | 0.22 | 1.564 | 61 | 1 | 132.1 | 135.4 | 10.8 | 0.0 | 278.3 | 364.0 | 0.51 | 0.43 | 0.06 | 0.32 | 0.76 |
| 34 | WI | 2.94 | 1.465 | 64 | 1 | 193.3 | 164.6 | 1.1 | 0.0 | 359.0 | 593.1 | 0.83 | 0.12 | 0.04 | 0.27 | 0.61 |
| 35 | WI | 1.14 | 1.527 | 59 | 1 | 181.6 | 176.8 | 8.3 | 0.0 | 366.7 | 481.0 | 0.68 | 0.24 | 0.09 | 0.14 | 0.76 |
| 36 | WI | 0.24 | 1.552 | 20 | 2 | 254.1 | 225.2 | 29.1 | 1.9 | 510.3 | 641.5 | 0.90 | 0.06 | 0.04 | 0.62 | 0.80 |
| 37 | WI | 0.97 | 1.663 | 30 | 1 | 92.7 | 149.7 | 8.2 | 0.0 | 250.6 | 427.9 | 0.60 | 0.27 | 0.13 | 0.14 | 0.59 |
| 38 | WI | 0.18 | 1.000 | 50 | 0 | 55.7 | 0.0 | 0.0 | 0.0 | 55.7 | 353.0 | 0.50 | 0.45 | 0.05 | 0.19 | 0.16 |
| 39 | WI | 0.08 | 1.172 | 4 | 1 | 41.1 | 8.5 | 0.0 | 0.0 | 49.6 | 206.1 | 0.29 | 0.65 | 0.06 | 0.02 | 0.24 |
| 40 | WI | 0.72 | 1.596 | 51 | 2 | 130.1 | 154.6 | 10.8 | 0.0 | 295.5 | 426.2 | 0.60 | 0.30 | 0.10 | 0.30 | 0.69 |
| 41 | WI | 0.62 | 1.486 | 62 | 1 | 194.1 | 181.7 | 0.6 | 0.0 | 376.4 | 577.0 | 0.81 | 0.15 | 0.04 | 0.48 | 0.65 |
| 42 | WI | 0.73 | 1.557 | 20 | 2 | 265.1 | 238.5 | 31.3 | 2.1 | 536.9 | 573.8 | 0.81 | 0.15 | 0.04 | 0.28 | 0.94 |
| 43 | WI | 0.48 | 1.304 | 30 | 1 | 182.7 | 69.8 | 4.0 | 0.0 | 256.6 | 369.4 | 0.52 | 0.34 | 0.14 | 0.28 | 0.69 |
| 44 | WI | 0.39 | 1.059 | 59 | 0 | 25.7 | 1.6 | 0.0 | 0.0 | 27.3 | 141.7 | 0.20 | 0.77 | 0.04 | 0.27 | 0.19 |
| 45 | WI | 0.72 | 1.332 | 69 | 1 | 286.9 | 135.1 | 3.1 | 0.0 | 425.1 | 580.5 | 0.81 | 0.15 | 0.04 | 0.20 | 0.73 |
| 46 | WI | 0.38 | 1.362 | 26 | 1 | 165.5 | 79.9 | 5.4 | 0.0 | 250.8 | 546.0 | 0.77 | 0.12 | 0.11 | 0.14 | 0.46 |
| 47 | WI | 0.35 | 1.647 | 14 | 2 | 99.5 | 83.3 | 26.0 | 0.1 | 208.8 | 224.4 | 0.32 | 0.71 | 0.04 | 0.29 | 0.93 |
| 48 | WI | 1.27 | 1.319 | 21 | 1 | 44.2 | 20.7 | 0.0 | 0.0 | 64.9 | 223.7 | 0.32 | 0.62 | 0.07 | 0.22 | 0.29 |
| 49 | WI | 0.50 | 1.633 | 14 | 0 | 26.3 | 45.5 | 0.0 | 0.0 | 71.8 | 341.1 | 0.48 | 0.48 | 0.04 | 0.11 | 0.21 |
| 50 | WI | 0.55 | 1.249 | 72 | 0 | 42.9 | 14.2 | 0.0 | 0.0 | 57.1 | 230.9 | 0.33 | 0.57 | 0.10 | 0.30 | 0.25 |

^*^Potato dominance metric (PDM)

^†^Total cultivated agriculture area was calculated from the sum of forage, fruit, maize, misc. crops, pea, potato, pulse, small grain, and vegetable area in each buffer.

^‡^Proportion potato in space was calculated as the proportion of cultivated cropland that grew potato in the year bioassays were conducted.

^§^Proportion potato in time was calculated as the proportion of cultivated cropland that grew potato in at least one of the four years preceding the year bioassays were conducted.
