# Supplementary material for: Spatial and Temporal Potato Intensification Drives Insecticide Resistance in the Specialist Herbivore, Leptinotarsa decemlineata
Source: PLoS One. 2015 Jun 1;10(6):e0127576. doi: 10.1371/journal.pone.0127576 (PMC4452079; doi:10.1371/journal.pone.0127576)
Supplement: S2 Dataset — Land cover estimates were tabulated from NASS CDL data using a 1.5 km buffer surrounding sample field centroids. (DOCX) [file pone.0127576.s002.docx]

| **ID** | **Forage** | **Fruit** | **Maize** | **Misc crops** | **Pea** | **Potato** | **Bean** | **Small grain** | **Vegetable** | **Forest** | **Grassland** | **Open water** | **Pasture** | **Urban** | **Wetland** |
| --- | --- | --- | --- | --- | --- | --- | --- | --- | --- | --- | --- | --- | --- | --- | --- |
| 1 | 26.5 | 0.0 | 75.1 | 4.0 | 37.6 | 72.7 | 40.4 | 103.6 | 0.0 | 176.8 | 8.7 | 4.0 | 55.4 | 43.5 | 63.4 |
| 2 | 25.2 | 0.0 | 176.4 | 3.6 | 0.0 | 97.8 | 27.4 | 36.6 | 1.6 | 218.3 | 9.3 | 1.4 | 49.9 | 43.8 | 20.3 |
| 3 | 39.1 | 0.0 | 176.9 | 13.6 | 0.0 | 299.1 | 16.6 | 5.4 | 0.4 | 56.8 | 28.6 | 1.5 | 26.2 | 34.3 | 12.9 |
| 4 | 23.8 | 0.1 | 47.4 | 11.2 | 0.1 | 237.9 | 31.7 | 151.6 | 0.1 | 45.7 | 10.0 | 0.2 | 76.6 | 42.5 | 32.7 |
| 5 | 54.1 | 0.1 | 5.8 | 5.8 | 65.2 | 145.2 | 5.1 | 33.7 | 0.1 | 171.2 | 10.1 | 0.6 | 53.3 | 44.7 | 116.5 |
| 6 | 48.2 | 0.0 | 30.9 | 8.6 | 89.3 | 19.7 | 28.1 | 132.2 | 0.0 | 88.6 | 9.4 | 0.3 | 43.8 | 31.2 | 180.7 |
| 7 | 4.4 | 0.0 | 175.5 | 6.8 | 0.0 | 49.5 | 165.6 | 19.6 | 8.7 | 108.4 | 1.8 | 14.5 | 38.5 | 3.0 | 116.8 |
| 8 | 22.6 | 0.7 | 129.0 | 0.4 | 6.7 | 48.1 | 42.9 | 82.9 | 0.3 | 151.8 | 2.7 | 29.5 | 31.2 | 22.4 | 140.9 |
| 9 | 68.8 | 0.0 | 143.0 | 2.2 | 8.5 | 145.7 | 16.0 | 7.6 | 2.5 | 149.3 | 12.5 | 6.0 | 34.9 | 26.4 | 88.9 |
| 10 | 32.8 | 0.1 | 175.5 | 8.7 | 0.5 | 140.3 | 25.2 | 6.7 | 2.4 | 122.5 | 78.9 | 1.9 | 55.3 | 39.6 | 19.9 |
| 11 | 101.1 | 0.1 | 182.6 | 6.7 | 1.5 | 157.0 | 22.1 | 6.1 | 3.0 | 91.3 | 57.6 | 2.6 | 16.3 | 31.9 | 26.9 |
| 12 | 21.2 | 0.0 | 211.1 | 0.0 | 0.0 | 67.3 | 150.4 | 23.9 | 8.6 | 102.2 | 3.2 | 0.2 | 61.0 | 38.8 | 20.5 |
| 13 | 19.9 | 0.0 | 74.6 | 1.8 | 4.6 | 93.9 | 73.6 | 10.3 | 87.4 | 183.6 | 48.4 | 0.5 | 20.7 | 62.1 | 30.2 |
| 14 | 17.7 | 0.0 | 38.1 | 1.1 | 48.4 | 161.4 | 22.2 | 34.9 | 86.4 | 138.0 | 48.8 | 0.0 | 34.4 | 68.9 | 12.8 |
| 15 | 1.4 | 0.0 | 64.2 | 0.1 | 3.2 | 105.4 | 62.5 | 2.6 | 168.7 | 173.5 | 64.9 | 6.3 | 16.5 | 34.4 | 8.4 |
| 16 | 11.9 | 0.0 | 115.5 | 0.4 | 14.4 | 123.7 | 27.0 | 27.3 | 93.5 | 138.6 | 24.3 | 5.1 | 29.6 | 84.3 | 16.4 |
| 17 | 24.6 | 0.0 | 89.3 | 0.7 | 41.5 | 109.1 | 73.2 | 48.7 | 175.2 | 22.9 | 13.2 | 0.2 | 40.0 | 64.6 | 8.5 |
| 18 | 10.3 | 0.0 | 72.8 | 0.7 | 38.9 | 54.3 | 95.7 | 42.3 | 35.1 | 92.2 | 84.0 | 0.9 | 32.4 | 140.6 | 11.6 |
| 19 | 5.0 | 0.0 | 152.9 | 0.0 | 34.5 | 82.8 | 154.0 | 25.4 | 156.1 | 31.3 | 8.7 | 0.0 | 3.5 | 51.7 | 5.8 |
| 20 | 15.4 | 0.0 | 14.8 | 2 | 0.1 | 36.42 | 15.96 | 18.6 | 61.0 | 275.1 | 44.8 | 10.3 | 65.5 | 128.2 | 23.1 |
| 21 | 2.5 | 0.0 | 194.7 | 0.0 | 2.3 | 154.0 | 14.9 | 2.0 | 0.2 | 178.2 | 32.4 | 0.4 | 67.6 | 57.0 | 5.8 |
| 22 | 10.7 | 0.0 | 193.1 | 0.0 | 3.1 | 91.2 | 74.4 | 23.8 | 0.4 | 21.4 | 13.9 | 0.0 | 213.0 | 51.9 | 13.5 |
| 23 | 2.3 | 0.0 | 244.7 | 0.0 | 15.6 | 29.1 | 64.3 | 4.1 | 3.4 | 131.1 | 19.8 | 0.0 | 97.0 | 95.0 | 5.8 |
| 24 | 3.1 | 0.0 | 226.1 | 0.0 | 41.8 | 81.3 | 83.5 | 2.5 | 3.1 | 76.9 | 23.8 | 0.0 | 91.2 | 75.9 | 3.2 |
| 25 | 7.4 | 0.0 | 193.7 | 0.0 | 1.8 | 43.1 | 100.9 | 1.2 | 0.0 | 156.2 | 54.0 | 0.4 | 81.6 | 66.8 | 5.6 |
| 26 | 65.4 | 0.0 | 264.5 | 0.0 | 0.9 | 37.0 | 88.4 | 4.3 | 4.9 | 124.7 | 8.6 | 0.0 | 43.6 | 65.9 | 4.0 |
| 27 | 31.9 | 0.0 | 125.0 | 0.0 | 0.4 | 160.1 | 40.2 | 2.9 | 41.3 | 190.3 | 20.4 | 0.0 | 38.2 | 56.6 | 5.4 |
| 28 | 53.9 | 0.0 | 137.8 | 0.0 | 23.2 | 140.1 | 204.2 | 24.7 | 3.4 | 18.1 | 7.8 | 0.0 | 40.7 | 57.7 | 0.7 |
| 29 | 66.1 | 0.0 | 139.8 | 0.0 | 14.0 | 159.0 | 80.4 | 39.1 | 3.1 | 99.6 | 12.2 | 0.2 | 21.3 | 72.0 | 4.7 |
| 30 | 36.8 | 0.0 | 110.3 | 0.6 | 12.4 | 233.2 | 83.0 | 14.4 | 2.7 | 116.8 | 10.3 | 0.1 | 48.2 | 37.3 | 5.8 |
| 31 | 51.3 | 0.1 | 138.1 | 0.4 | 0.0 | 33.9 | 10.5 | 5.8 | 0.0 | 261.1 | 34.8 | 22.8 | 58.2 | 50.7 | 40.3 |
| 32 | 76.7 | 0.1 | 116.2 | 0.1 | 0.1 | 22.1 | 44.2 | 2.7 | 0.7 | 207.8 | 17.8 | 0.3 | 61.6 | 27.5 | 132.0 |
| 33 | 30.4 | 0.0 | 175.3 | 0.1 | 0.0 | 115.6 | 36.4 | 4.5 | 1.6 | 225.6 | 37.3 | 0.1 | 34.0 | 41.9 | 9.4 |
| 34 | 22.8 | 1.3 | 237.2 | 0.4 | 9.8 | 162.2 | 152.6 | 6.0 | 0.7 | 37.2 | 15.9 | 3.5 | 29.4 | 30.3 | 2.5 |
| 35 | 15.7 | 0.0 | 261.4 | 0.2 | 3.4 | 68.9 | 123.7 | 5.4 | 2.2 | 81.4 | 17.0 | 5.9 | 55.8 | 60.8 | 10.0 |
| 36 | 27.3 | 0.0 | 73.9 | 0.1 | 0.2 | 400.0 | 89.3 | 50.7 | 0.2 | 19.0 | 6.1 | 0.0 | 15.1 | 29.9 | 0.7 |
| 37 | 29.4 | 0.8 | 150.1 | 0.1 | 1.0 | 60.8 | 160.1 | 10.3 | 15.1 | 93.6 | 50.7 | 0.2 | 41.3 | 90.9 | 7.0 |
| 38 | 61.8 | 0.0 | 140.4 | 0.9 | 0.1 | 67.5 | 73.4 | 8.9 | 0.0 | 91.4 | 4.9 | 0.5 | 96.7 | 38.1 | 124.7 |
| 39 | 7.9 | 0.2 | 128.0 | 19.0 | 0.3 | 3.4 | 24.3 | 3.4 | 19.6 | 255.2 | 41.0 | 74.5 | 29.7 | 40.9 | 64.8 |
| 40 | 20.4 | 0.0 | 95.8 | 0.1 | 7.9 | 127.5 | 66.1 | 2.3 | 105.9 | 118.2 | 32.0 | 5.5 | 32.7 | 69.1 | 28.4 |
| 41 | 32.2 | 0.4 | 128.6 | 1.6 | 0.0 | 279.4 | 101.6 | 13.5 | 19.7 | 28.6 | 32.7 | 0.8 | 36.8 | 30.7 | 4.9 |
| 42 | 16.5 | 0.0 | 235.5 | 0.1 | 6.7 | 162.8 | 123.3 | 28.9 | 0.1 | 22.9 | 18.8 | 0.0 | 64.6 | 29.4 | 2.8 |
| 43 | 13.0 | 0.0 | 100.4 | 0.3 | 0.2 | 102.8 | 139.5 | 12.8 | 0.4 | 100.3 | 77.3 | 0.0 | 56.6 | 98.3 | 9.4 |
| 44 | 21.5 | 0.5 | 14.8 | 8.7 | 0.0 | 38.1 | 9.0 | 1.3 | 47.7 | 255.5 | 54.7 | 29.7 | 13.4 | 25.3 | 192.0 |
| 45 | 43.7 | 0.3 | 275.9 | 0.0 | 1.8 | 117.9 | 138.7 | 2.2 | 0.0 | 54.3 | 8.0 | 0.0 | 37.9 | 27.9 | 4.1 |
| 46 | 64.6 | 0.0 | 198.4 | 0.2 | 0.0 | 74.4 | 181.1 | 24.2 | 3.1 | 37.3 | 27.0 | 0.0 | 19.3 | 81.5 | 0.8 |
| 47 | 84.3 | 0.0 | 53.7 | 13.5 | 0.0 | 64.8 | 1.3 | 6.8 | 0.0 | 115.9 | 2.5 | 56.0 | 16.8 | 26.5 | 266.0 |
| 48 | 63.1 | 0.0 | 81.5 | 0.2 | 0.0 | 48.5 | 30.1 | 0.4 | 0.0 | 244.4 | 2.1 | 51.2 | 28.5 | 46.3 | 113.9 |
| 49 | 52.6 | 0.1 | 186.8 | 10.3 | 1.1 | 38.2 | 41.7 | 10.3 | 0.1 | 109.9 | 8.5 | 1.6 | 51.9 | 30.6 | 165.7 |
| 50 | 17.4 | 0.0 | 112.6 | 0.2 | 0.0 | 69.3 | 11.2 | 20.2 | 0.0 | 150.8 | 14.8 | 19.6 | 76.9 | 74.2 | 141.0 |
