# Supplementary figures and images for: Spatial and Temporal Potato Intensification Drives Insecticide Resistance in the Specialist Herbivore, Leptinotarsa decemlineata
Source: PLoS One. 2015 Jun 1;10(6):e0127576. doi: 10.1371/journal.pone.0127576 (PMC4452079; doi:10.1371/journal.pone.0127576)

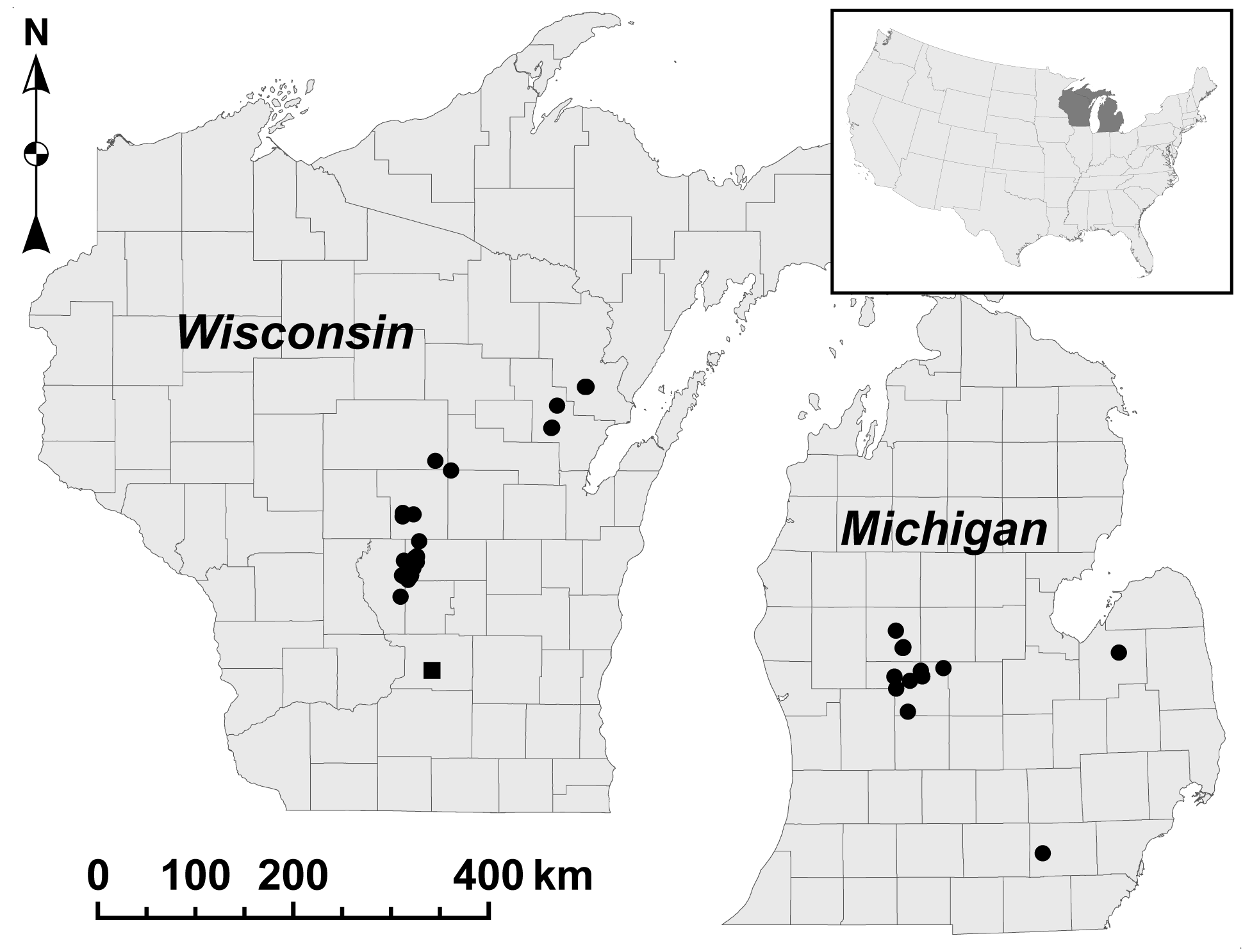

Supplement: S1 Fig — Map of L. decemlineata populations (N = 50) assayed for neonicotinoid resistance from 2007 to 2012. At each location, adult L. decemlineata were sampled from commercial potato fields and exposed to a dose-response bioassay. Circle (●) data points represent collections at commercial potato fields and the triangle (◼) represents reference population collection. (TIF) [file pone.0127576.s003.tif]

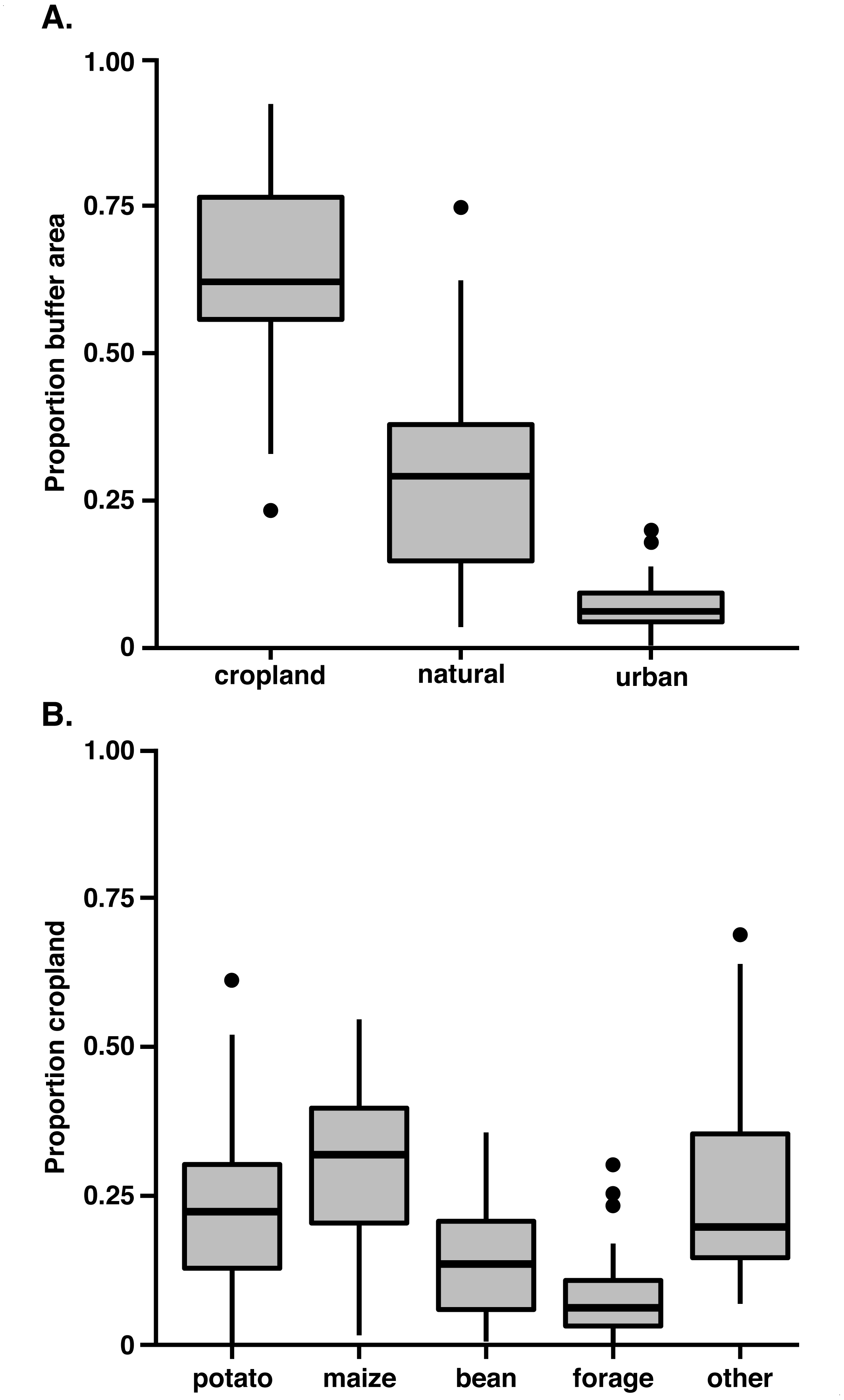

Supplement: S2 Fig — Distribution of major land cover types (a) comprising average proportion of 1.5 km radius surrounding sample field centroid. Distribution of dominant agricultural crop types comprising the average proportion cropland (b) of 1.5 km radius surrounding sample field centroid. Land cover compositions were measured in each year of L. decemlineata bioassay (N = 50 fields). Minor crops (i.e., fruit, miscellaneous crops, pea, small grains, and other vegetables) were aggregated for graphical presentation. (TIF) [file pone.0127576.s004.tif]

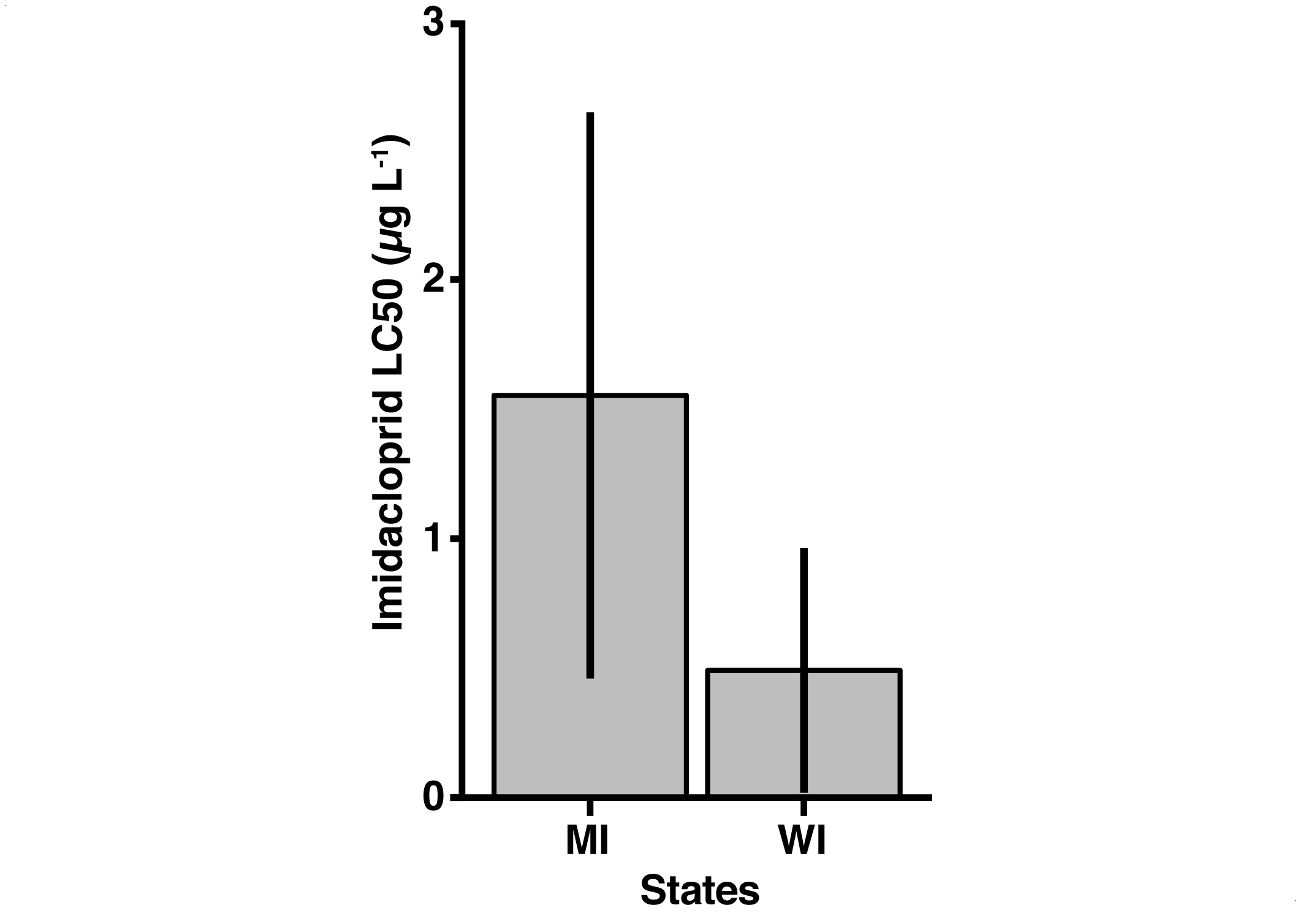

Supplement: S3 Fig — Average lethal concentration responses of L. decemlineata beetle populations to imidacloprid by state from 2007 to 2012. Error bars represent standard deviation of means. Lethal concentration estimates were significantly different between states (Student’s t-test, t = 3.2589, df = 12.467, P = 0.0065). (TIF) [file pone.0127576.s005.tif]

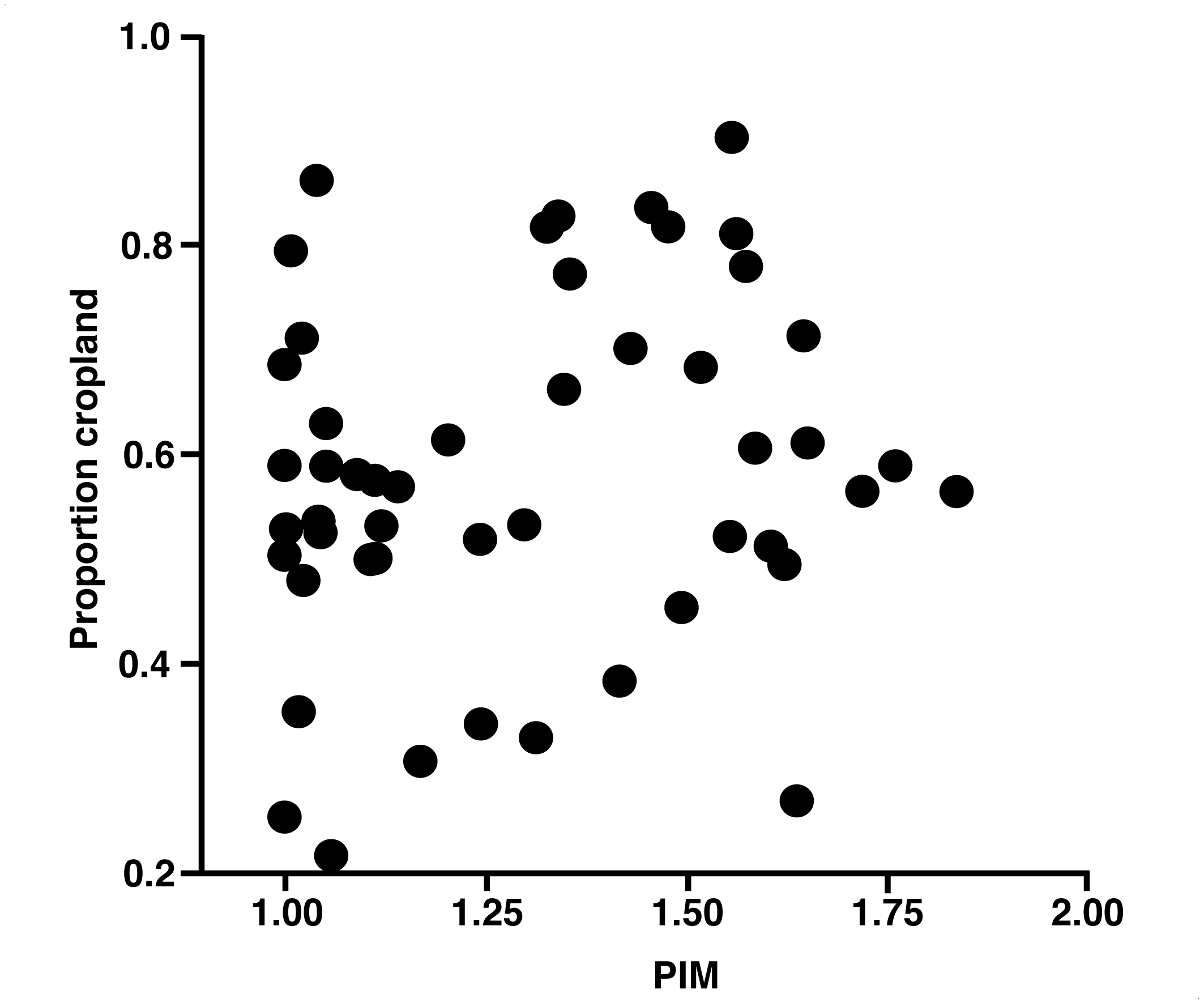

Supplement: S4 Fig — Relationship between the potato intensity metric (PIM) and proportion cropland in the landscape (N = 50 fields). Measures of proportion cropland and PIM were not significantly correlated. (TIF) [file pone.0127576.s006.tif]

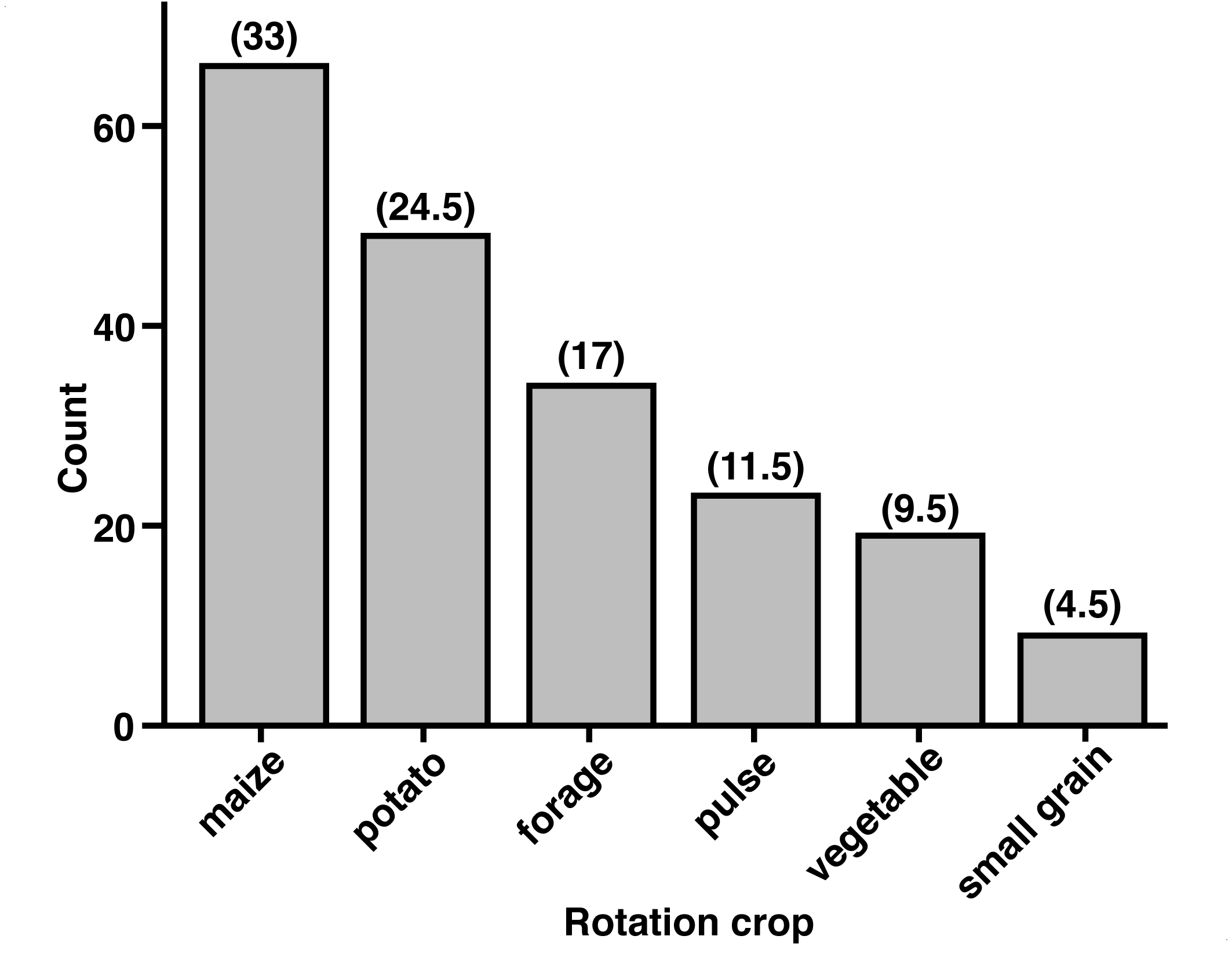

Supplement: S5 Fig — Frequency distribution of major crop groups grown in four prior seasons at 50 different field locations where L. decemlineata populations were assayed for neonicotinoid resistance from 2007 to 2012. Numbers in parentheses indicate the percentage of total counts for each group. (TIF) [file pone.0127576.s007.tif]
