# Supplementary material for: Spatial and Temporal Potato Intensification Drives Insecticide Resistance in the Specialist Herbivore, Leptinotarsa decemlineata
Source: PLoS One. 2015 Jun 1;10(6):e0127576. doi: 10.1371/journal.pone.0127576 (PMC4452079; doi:10.1371/journal.pone.0127576)
Supplement: S1 Table — (DOCX) [file pone.0127576.s008.docx]

| **Parameter** | **Estimate** | **Standard error** | ***t*-value** | **p-value** |
| --- | --- | --- | --- | --- |
| intercept*_michigan_* | -1.2804 | 0.6205 | -2.063 | 0.0446 |
| PIM | 1.0453 | 0.4075 | 2.565 | 0.0136 |
| state*_wisconsin_* | -1.0508 | 0.2421 | -4.341 | 0.0000757 |
| Model evaluation |  |  |  |  |
| Residual standard error | 0.6967 |  |  |  |
| Residual d.f. | 47 |  |  |  |
| Multiple R^2^ | 0.4297 |  |  |  |
| F-statistic | 17.71 |  |  |  |
| *P* value | 0.000001853 |  |  |  |
